# Successful reconstruction of the rat ureter by a syngeneic collagen tube with a cardiomyocyte sheet

**DOI:** 10.1016/j.reth.2023.10.001

**Published:** 2023-10-13

**Authors:** Shutaro Yamamoto, Kenji Matsui, Yoshitaka Kinoshita, Hiroshi Sasaki, Hidekazu Sekine, Yatsumu Saito, Yasuhide Nakayama, Haruki Kume, Takahiro Kimura, Takashi Yokoo, Eiji Kobayashi

**Affiliations:** a Department of Urology, The Jikei University School of Medicine, Tokyo 105-8461, Japan; b Division of Nephrology and Hypertension, Department of Internal Medicine, The Jikei University School of Medicine, Tokyo 105-8461, Japan; c Department of Urology, Graduate School of Medicine, The University of Tokyo, Tokyo 113-8654, Japan; d Institute of Advanced Biomedical Engineering and Science, Tokyo Women's Medical University, Tokyo 162-8666, Japan; e Osaka Laboratory, Biotube Co., Ltd., Osaka, 565-0842, Japan; f Department of Kidney Regenerative Medicine, The Jikei University School of Medicine, Tokyo 105-8461, Japan

**Keywords:** Biotube, Cardiomyocyte sheets, Regeneration, Scaffold, Ureter, Tissue engineering

## Abstract

**Introduction:**

Ureteral injuries require surgical intervention as they lead to loss of renal function. The current reconstructive techniques for long ureteral defects are problematic. Consequently, this study aimed to reconstruct the ureter in a rat model using subcutaneously prepared autologous collagen tubes (Biotubes).

**Methods:**

The lower ureter of LEW/SsNSlc rats was ligated to dilate the ureter to make anastomosis easier, and reconstruction was performed six days later by anastomosing the dilated ureter and bladder with a Biotube that was prepared subcutaneously in syngeneic rats. Some rats underwent left nephrectomy and ureter reconstruction simultaneously as negative controls to evaluate the effects of urine flow on patency. The other rats were divided into three groups as follows: a group in which the ureter was reconstructed with the Biotube alone, a group in which cardiomyocyte sheets made from the neonatal hearts of syngeneic rats were wrapped around the Biotube, and a group in which an adipose-derived stem cell sheets made from the inguinal fat of adult syngeneic rats were wrapped. Contrast-enhanced computed tomography and pathological evaluations were performed two weeks after reconstruction.

**Result:**

In the Biotube alone group, all tubes were occluded and hydronephrosis developed, whereas the urothelium regenerated beyond the anastomosis when the left kidney was not removed, suggesting that urothelial epithelial spread occurred with urinary flow. The patency of the ureteral lumen was obtained in some rats in the cardiomyocyte sheet covered group, whereas stricture or obstruction of the reconstructed ureter was observed in all rats in the other groups. Pathological evaluation revealed a layered urothelial structure in the cardiomyocyte sheet covered group, although only a small amount of cardiomyocyte sheets remained.

**Conclusion:**

Urinary flow may support the epithelial spread of the urothelium into the reconstructed ureter. Neonatal rat cardiomyocyte sheets supported the patency of the regenerated ureter with a layered urothelium.

## Highlights

• Syngeneic collagen tubes were used for the reconstruction of the rat ureter.
• The presence of urine flow may promote the regeneration of urothelium.
• Covering a cardiomyocyte sheets improved patency of Biotube.
• Novel strategy of ureteral reconstruction was presented.

## 1 Introduction

Ureteral injuries may lead to the loss of renal function and hence require early surgical intervention. Generally, 75–90 % of ureteral injuries are iatrogenic, with intraoperative injuries being common [1]. Although minimally invasive surgery for malignant tumors has advanced in recent years with the introduction of laparoscopic and robotic surgery, it is reportedly more prone to intraoperative ureteral injury, mainly in the lower ureter [2].

If the injury is short, end-to-end ureteral anastomosis can be performed; nevertheless, if the lesion is long, urinary diversion such as “ileal ureteral substitution,” “ileal conduit,” “cutaneous ureterostomy” or “nephrostomy” is selected [1]. However, there are many problems to be solved with these procedures, including increased susceptibility to infection and decreased quality of life [3,4].

Bioengineered ureters have been actively studied to solve these problems. Although some attempts have been made to use decellularized blood vessels or GoreTex tubes as scaffolds and seed cells such as urothelial cells or mesenchymal stem cells, there are still issues in clinical applications, including the inability to induce peristalsis of the ureters [1,5]. Recently, the autologous collagen tube (Biotube; Biotube Co., Ltd., Tokyo, Japan) using in-body tissue architecture technology has been under clinical investigation in the field of vascular grafts. The fibroblasts invade and form an autologous collagen tube after a certain period by implanting a cylindrical mold under the skin. When the tubes are anastomosed with vessels, they promote endothelial and smooth muscle regeneration, allowing long-term lumen patency [[6], [7], [8]]. Moreover, it has been shown that patchy implants can reconstruct tissues such as heart valves, trachea, and bladder. They have been reported to be functional as a scaffold for autologous tissue regeneration [[6], [7], [8], [9], [10], [11], [12]]. Biotubes can be prepared from autologous tissues to avoid rejection, which is considered an advantage in clinical applications; hence, we focused on this material and hypothesized that it could be applied as a scaffold for ureteral reconstruction.

Consequently, this study aimed to investigate whether Biotubes could be used for ureter tissue engineering and to examine the effects of enveloping various cell sheets on tissue construction and patency.

## 2 Methods

### 2.1 Experimental animals and ethics

LEW/SsNSlc rats (LEW rats) purchased from Nihon SLC Inc. (Shizuoka, Japan) were used in all experiments. Adult eight weeks old female LEW rats were used for Biotube preparation and ureteral reconstruction experiments. One-day-old LEW rat neonates were used as donors for cardiomyocyte sheet preparation. Adult 4–6 weeks old male LEW rats were used to isolate adipose-derived stem cells (ASCs). The experiments were performed following the Guide for the Care and Use of Laboratory Animals published by the United States National Institutes of Health (NIH Publication No. 85-23, revised 1996) [13]. All the experiments were approved by the Jikei University Ethics Committee (No. 2020-077).

### 2.2 Preparation of Biotube

Biotubes were prepared as previously described [6]. Briefly, adult LEW rats (male) were anesthetized with isoflurane (5 % induction, 2 % maintenance), a small incision was made in the back, and a Biotube mold, provided from Biotube Co., Ltd. (wall thickness approximately 0.4 mm, cylinder 2 mm in diameter and 20 mm in length), was inserted through the incision. The incision was sutured with a 5-0 silk thread. The mold was embedded at two sites on the left and right sides.

One month after mold implantation, the rats in which the molds were implanted were anesthetized, and the molds were removed through an incision. Thereafter, the rats were euthanized. The tissue around the mold was carefully detached, and a tube made of collagen fibers that formed between the inner and outer molds was removed and stored at room temperature in 70 % ethanol until use.

### 2.3 Preparation of cardiomyocyte sheets

Cardiomyocytes were isolated from the ventricles of 1-day-old LEW rats and cultured as previously described [14]. Briefly, the ventricles were shredded with a new scalpel in Hanks' solution and washed twice with a dish in new Hanks' solution, then collected in a triangular flask, and 15 mL of Hanks' solution with type II collagenase (0.06 %; Sigma, Lakewood, NJ, USA) was added and incubated at 37 °C for 10 min. Subsequently, the supernatant was removed and added to a 50-mL tube containing 5 mL of Hanks' solution + fetal bovine serum (FBS) (1:1) to inhibit the reaction. The same procedure was repeated thrice for the remaining cardiac tissue, and the solution in the four tubes was centrifuged (3000 rpm for 10 min). Sedimented cardiomyocytes were collected into Dulbecco's modified Eagle's medium (DMEM) high-glucose + M199 medium (1:1) + 6 % FBS + 1 % Penicillin-Streptomycin solution (PS) (Culture medium, CM), and passed through a 40 μm cell strainer (Becton Dickinson, Franklin Lakes, NJ, USA). Cells were seeded at 3 × 10ˆ6 cells/well in CM into 35 mm temperature-responsive culture dishes (CellSeed, Tokyo, Japan) and kept at 37 °C in an incubator. The CM was changed every alternate day. After four days, the temperature-responsive culture dishes were moved to room temperature, and the cell sheets detached spontaneously from the culture dishes. Next, the cell sheets were formed into a three-layered structure. The sheets produced were used immediately for transplantation experiments.

### 2.4 Preparation of ASC sheets

ASCs were isolated from the inguinal adipose tissue of adult LEW rats, as previously reported [15]. Inguinal adipose tissue was cut into small pieces, digested with type II collagenase (0.2 %) for 3 h at 37 °C, and then isolated as a single cell suspension through a 40 μm cell strainer. After centrifugation at 700*g* for 5 min, the cells were collected, plated on a 100-cm^2^ culture dish, and cultured at 37 °C in a humid 5 % CO_2_ incubator. When the cells reached 70–90 % confluence, they were harvested using a 0.25 % trypsin-EDTA solution (Nacalai Tesque Inc.) and cryopreserved. Subsequently, the cryopreserved ASCs were thawed and seeded at 5–10 × 10ˆ5 cells/well in 35 mm temperature-responsive culture dishes (CellSeed, Tokyo, Japan) with DMEM F12 + 10 % FBS + 1 % PS and cultured in an incubator (37 °C, 5 % CO_2_). Five days later, the cells spontaneously detached from the temperature-responsive dish and formed a sheet when transferred to room temperature. Subsequently, cell sheets were formed into three-layered structures, which were used immediately for transplantation experiments.

### 2.5 Preparation of ureteral obstruction rat model

The abdomen was opened via a midline abdominal incision under general anesthesia. The retroperitoneum was incised, and the left ureter was exposed and ligatured using a 10-0 nylon thread in the lower one-third position. The muscular layer and skin were sutured with a 5-0 silk thread, and the abdomen was closed.

### 2.6 Ureteral reconstruction with Biotubes

Six days after ureteral ligation, rats with ureteral dilation were used. The abdomens of the rats were opened under anesthesia, and the dilated left ureter was identified. The dilated ureter was cut above the ligation site and anastomosed end-to-end with a Biotube using eight interrupted 7-0 nylon sutures. The bladder was incised, the bladder and Biotube were anastomosed similarly, and the Biotube replaced 7 mm, 1 cm, and 2 cm long ureters. In the Biotube alone group, the abdomen was closed, whereas the sheets were covered in the group with cardiomyocytes and ASC sheets.

### 2.7 Preparation of left nephrectomy rat model

The effect of urine flow on urothelial regeneration was investigated by creating a model in which ureteral reconstruction and left nephrectomy were simultaneously performed to eliminate urinary flow. After the ureter was reconstructed using a Biotube, the left renal artery and vein were ligated using a 5-0 silk thread. Moreover, the ureter was ligated above the anastomosis with the Biotube, and the left kidney was removed. The procedure was completed by suturing the muscle layers and the skin.

### 2.8 Covering with cardiomyocyte and ASC sheets

After ureteral reconstruction with the Biotubes, three-layered cardiomyocyte and ASC sheets were wrapped around the reconstructed ureter in the cardiomyocyte and ASC sheet groups, respectively, to cover the entire length of the Biotubes; one set of three-layered sheets was used for 7 mm or 1 cm replacement and two sets of three-layered sheets for 2 cm replacement. The surrounding tissue was covered outside the sheets to prevent the separation of the Biotubes and their respective sheets. The procedure was completed by suturing the muscle layers and the skin.

### 2.9 Evaluation of patency

The patency of the replaced ureter in rats whose ureters were reconstructed with Biotubes alone, Biotubes with cardiomyocyte sheets, and Biotubes with ASC sheets, was assessed with contrast-enhanced computed tomography (CT). Rats were anesthetized with a 5 mL/kg intraperitoneal injection of a mixture of medetomidine (75 g/mL; Meiji Seika Pharma Co., Tokyo, Japan), butorphanol (0.5 mg/mL; Meiji Seika Pharma Co., Tokyo, Japan) and midazolam 0.4 mg/mL (Yamanouchi, Tokyo. Japan) 5 mL/kg. Iopamidol 300 (2.7 mL/kg; Bayer Pharma Japan, Osaka, Japan) was injected intravenously (twice at 0 and 25 min) through the penile vein, and CT (LCT-200, Nippon RayTech Co., Ltd., Tokyo, Japan) was performed 30 min later to confirm the patency of the replaced ureter, which was classified as a luminal patency, stricture, or obstruction. ‘Luminal Patency’ was defined as a state in which the ureter was open and no hydronephrosis was observed; ‘Stricture’ was defined as a state in which the ureter was open but hydronephrosis was observed; and ‘Obstructed’ was defined as a state in which the ureter was not open.

### 2.10 Immunohistochemistry evaluation

Specimens were fixed in 4 % paraformaldehyde (Wako, Osaka, Japan) in phosphate-buffered saline (PBS) overnight, dehydrated in 15 % sucrose in PBS overnight, and in 30 % sucrose in PBS overnight at 4 °C. Specimens were embedded in the OCT compound (Sakura Finetek, Tokyo, Japan), and 10-μm thick frozen sections were prepared. Antigen retrieval for immunostaining was performed using 10 % HistoVT One/PBS (Nacalai Tesque, Kyoto, Japan) in a warm bath at 70 °C for 20 min. After blocking with Blocking One Histo (Nacalai Tesque) for 10 min at room temperature, the sections were incubated with primary antibodies overnight at 4 °C. Thereafter, the sections were incubated with secondary antibodies for 1 h at room temperature, consecutively conjugated to Alexa Fluor 488, 546, or 647 for 1 h at room temperature. Sections were mounted with Prolong Gold Antifade Mountant with 4′,6-diamidino-2-phenylindole (DAPI) (P36931, Thermo Fisher Scientific). Each sample was examined under a fluorescence microscope (LSM880 confocal; Carl Zeiss, Munich, Germany). Regarding the primary antibodies, we used anti-GATA binding protein 3 (GATA3; AF2605, R&D systems, Minneapolis, MN, USA), anti-tumor protein 63 (TP63; GTX102425, GeneTex, Irvine, CA, USA), anti-alpha smooth muscle actin (αSMA; A2547, Sigma Aldrich, St. Louis, MO, USA), anti-CD31 (AF3628; R&D systems, Minneapolis, MN, USA), anti-cytokeratin 8 (CK8; ab53280, Abcam, Cambridge, UK), anti-uroplakin 3 (UP3; ab78196, Abcam), and anti-cardiac troponin T (cTnT; MS-295-P, Thermo Fisher Scientific).

## 3 Results

### 3.1 Biotube alone

**Fig. 1:**
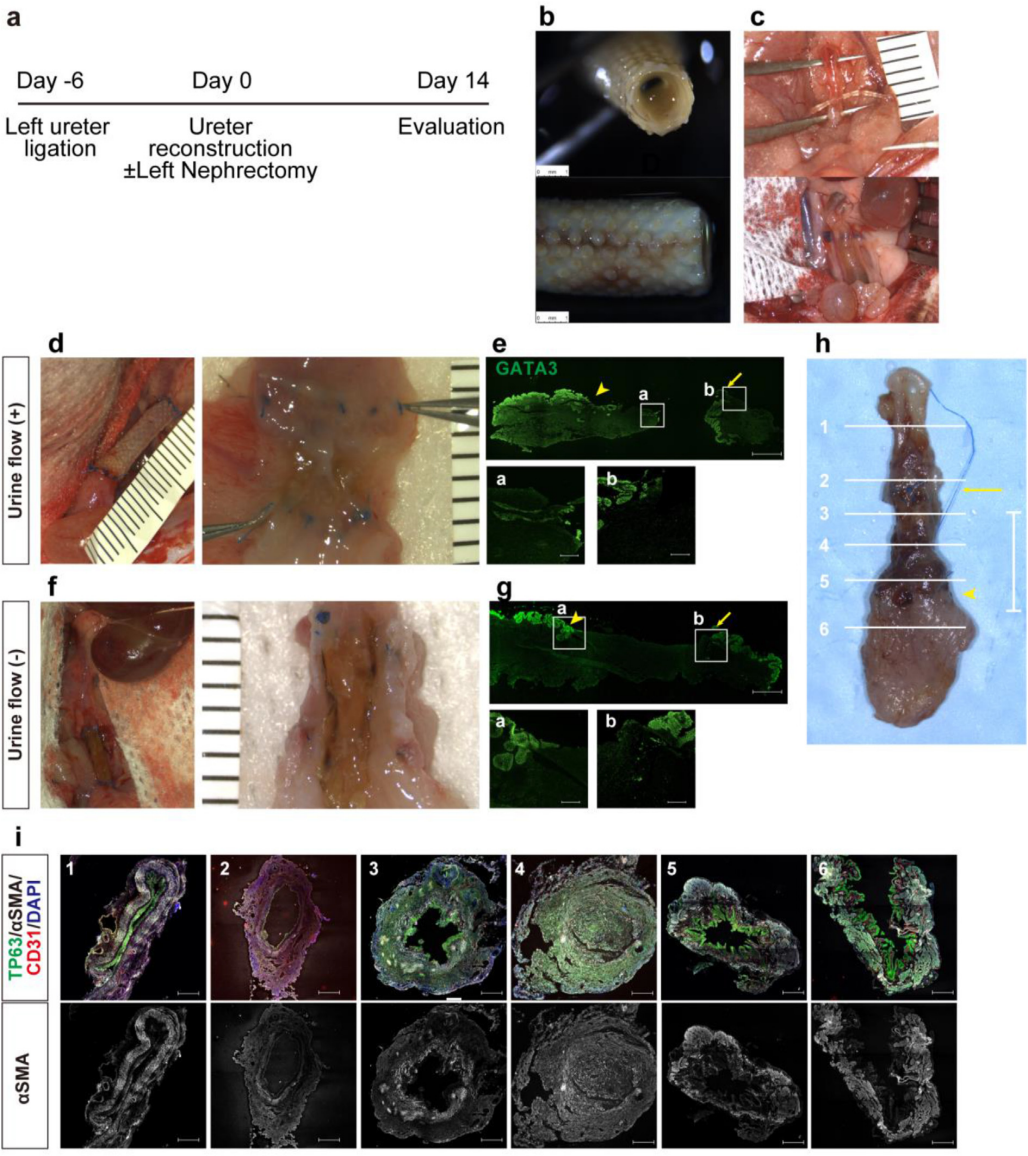
Experimental protocol and pathological evaluation of ureteral reconstruction with collagen tube alone with or without left nephrectomy. (a) The schedule of left ureteral ligation, ureteral reconstruction surgery with or without left nephrectomy, and evaluation. (b) Gross image of collagen tube retrieved 1 month after mold implantation. (c) Gross images of the left ureter of the rat before (upper panel) and 6 days after (lower panel) ureteral ligation. (d and f) The appearance of the collagen tube connecting the host left ureter and bladder at the time of reconstruction (left panel) and the lumenal surface of the reconstructed ureter at the time of specimen retrieval (right panel) in a subject that underwent ureteral reconstruction surgery without nephrectomy (d) or with nephrectomy (f), using a collagen tube alone. Tissue spread beyond the anastomosis on the lumen surface was observed only in (d). (e and g) Immunostaining using GATA3 of (d) and (f), respectively. Anastomoses at the caudal and cephalic sides are marked by yellow arrowheads and arrows, respectively. Scale bars: 500 μm (bottom). 1 mm (above) severally. (h) A gross image of a specimen in which ureteral reconstruction was performed using a collagen tube alone. Sections were made at sites 1–6 to include the area around the anastomosis (caudal: arrowhead, cephalic: arrow). Scale bars: 10 mm. (i) Immunostaining with TP63, αSMA, CD31, and DAPI of (h) at 1–6 each. Scale bars: 200 μm. GATA3, GATA-binding protein 3; TP63, tumor protein 63; αSMA, alpha-smooth muscle actin.

**Fig. 2:**
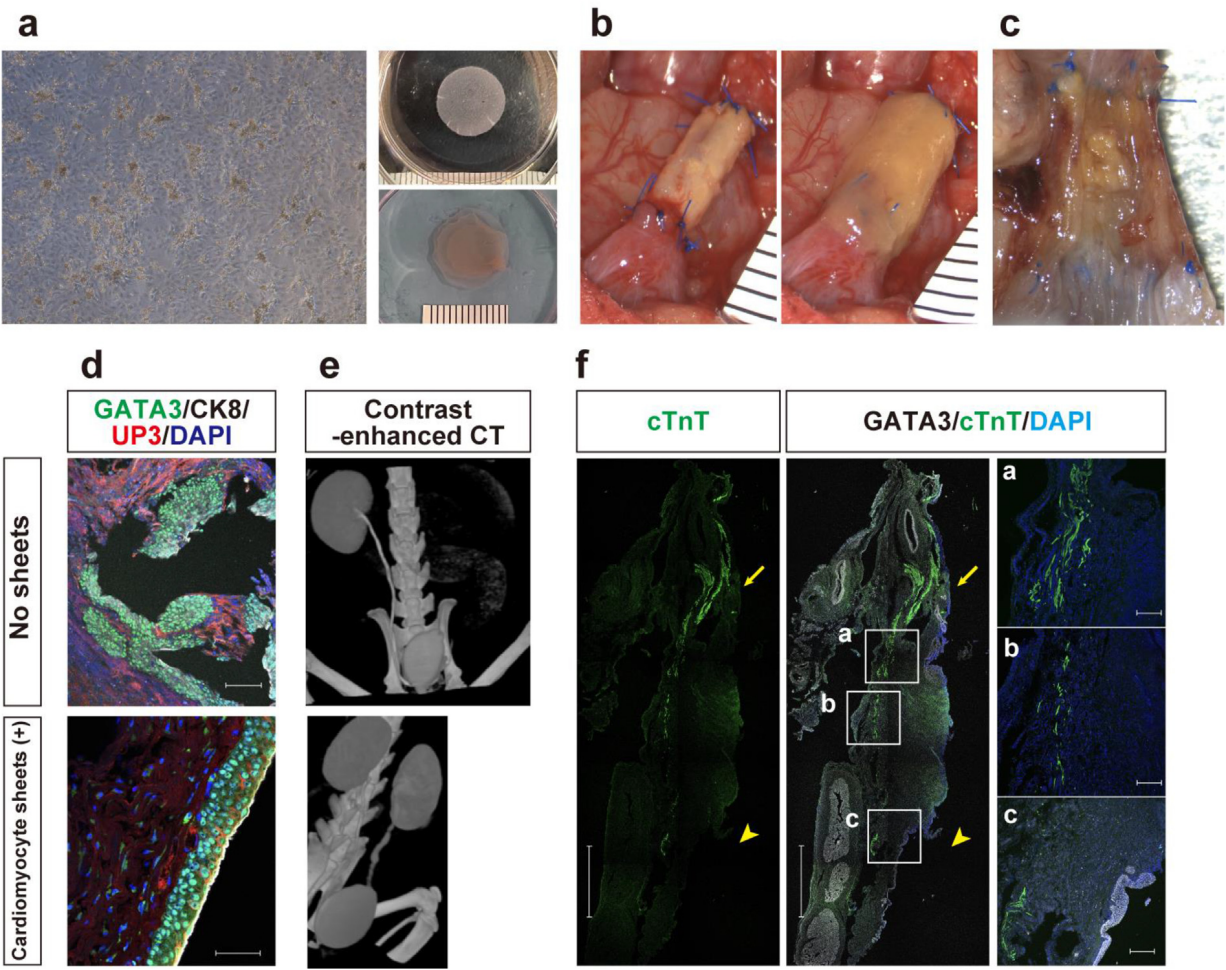
Evaluation of the cardiomyocyte sheet group. (a) After 4 days of culture, cardiomyocytes (left) were cooled to room temperature, where they detached from the dish and shrank to form sheets (upper right). Three sheets were stacked (bottom right) and used for transplantation (b) after reconstruction using the collagen tube (left) and covered with the cardiomyocyte sheet (right). (c) The luminal surface of the reconstructed ureter 2 weeks after the anastomosis, exposed through a longitudinal incision from the ventral side. (d) Immunostaining of all layers of uroepithelium (GATA3) and superficial cells (UP3) on the reconstructed ureters of the collagen tube alone (above) and with the cardiomyocyte sheet (below). Scale bars: 200 μm (above), 50 μm (below) for each. (e) Contrast-enhanced CT images of the collagen tube alone (above) and with the cardiomyocyte sheet (below). There is no hydronephrosis, and the ureter is open in the subject with the cardiomyocyte sheet. (f) Immunostaining of reconstructed ureters retrieved from subjects with cardiomyocyte sheet including the cephalic anastomosis (arrow) and caudal anastomosis (arrowhead), stained for cTnT and urothelium (GATA3). Only minimal residual cardiomyocyte tissue was observed. Lumen is on the right side. Scales 2 mm (two on the left), 200 μm (Three in a vertical line on the right). (GATA3: GATA-binding protein 3, UP3: Uroplakin 3, cTnT: cardiac troponin T).

The protocol for ureteral reconstruction using the Biotubes fabricated from LEW rats is shown in Fig. 1a. When the mold was implanted subcutaneously in LEW rats in advance and retrieved after one month, an elastic Biotube was formed (Fig. 1b). The lower left ureter was ligated (Fig. 1c, top) and dilated (Fig. 1c, bottom) in advance to reproduce ureteral dilation due to ureteral injury and facilitate anastomotic surgery. The ureter was reconstructed with a Biotube (Fig. 1d, left). Two weeks later, the middle part of the Biotube was occluded, and hydronephrosis developed. However, tissue growth was observed in the lumen beyond the anastomoses at both ends (Fig. 1d, right). Immunostaining revealed that the urothelium stained with GATA3 covered the lumen of the reconstructed ureter (Fig. 1e). In the control group that underwent left nephrectomy and reconstruction to stop the urinary flow (Fig. 1f, left), no urothelial spread was observed (Fig. 1f, right and 1g). A transverse section was stained (Fig. 1h) to confirm the cause of obstruction in the Biotube alone group. In addition to the host ureter (Fig. 1h–) and host bladder (Figs. 1h–6), an array of urothelial cells can be seen at the upper-end (Fig. 1, Fig. 2; yellow arrowhead) and lower-end anastomoses (Figs. 1h–5; yellow arrowhead). Moreover, slightly below the upper-end anastomosis, urothelial cells and SMA-positive myofibroblasts existed (Figs. 1h–3). However, the site of obstruction lacked urothelial layers and showed a disorganized proliferation of myofibroblasts. (lower Figs. 1h–4).

### 3.2 Cardiomyocyte sheet

The ventricles of the LEW neonates were collected and single-celled to form sheets. After four days of culture, the cardiomyocytes began to beat (Fig. 2a, left) and were made into three-layered sheets (Fig. 2a, right), which were then enveloped around the reconstructed ureter (Fig. 2b). Subjects with a patent reconstructed ureter were obtained two weeks later in this population (Fig. 2c). Even in the reconstructed area, GATA3-positive urothelium was aligned with UPK3-positive superficial cells on the surface layer, maintaining the laminar structure of the urothelium. However, this arrangement was disorganized in the Biotube alone group (Fig. 2d). Patency was confirmed using contrast-enhanced CT (Fig. 2e). The patency rate improved compared to the Biotube alone group (Table 1). However, peristalsis or beating of the regenerated ureter was not confirmed, and few cardiomyocytes remained (Fig. 2f).

**Table 1:** The results of reconstruction surgeries in three groups.

	No Sheets			Cardiomyocyte sheets			ASC sheets		
	Luminal Patency	Stricture	Obstruction	Luminal Patency	Stricture	Obstruction	Luminal Patency	Stricture	Obstruction
7 mm, 2 weeks	–	–	–	1	0	0	–	–	–
1 cm, 2 weeks	0	0	3	0	1	1	0	1	1
2 cm, 2 weeks	0	0	1	1	0	0	0	0	3
Total	0	0	4	2	1	1	0	1	4

ASC, adipose-derived stem cell.

### 3.3 Adipose-derived stem cell sheet

To test whether the improvement in patency with the cardiomyocyte sheet was due to the secretion of humoral factors rather than movement, ASC sheets that did not move but secreted humoral factors were wrapped around Biotubes instead of cardiomyocyte sheets. However, none of the subjects in the ASC sheet had the patency of the ureteral lumen (Table 1). Pathological examination revealed an increase in SMA-positive myofibroblasts in the occluded area.

## 4 Discussion

In this study, we examined the effects of Biotube with cardiomyocyte sheet or ASC sheet on the reconstruction of an obstructed ureter. The cardiomyocyte sheet group achieved lumen patency and pathologically well layered urothelial regeneration. In the rats ofreconstruction with the Biotube alone, the urothelial spread in the lumen of the Biotube was observed only in rats with preserved urinary flow, suggesting that the presence of urinary flow is necessary for urothelial spread. However, in all cases of reconstruction using Biotubes alone, the reconstructed ureter was completely occluded, and there was an uncontrolled proliferation of myofibroblasts in the occluded area. Although the Biotubes show good patency in high-pressure systems such as arteries [[6], [7], [8]], in the ureter, which is a low-pressure system, the reconstructed structures seemed to be occluded by myofibroblast proliferation before urothelial spread due to poor intra-luminal urine flow. We focused on peristalsis as a ureteral function that maintains ureteral urinary flow. Ureteral peristalsis is governed by primary pacemaker cells (atypical smooth muscles) at the renal pelvis-kidney junction, and synchronized peristalsis occurs when electrical signals are transmitted between contacting smooth muscle cells [[16], [17], [18], [19]]. In the absence of atypical smooth muscle, specialized interstitial cells in the ureteric interstitium supplement the pacemaker function [6]. Basic studies have shown that mice lacking the genes involved in the development of pacemaker cells (sonic hedgehog (SHH), angiotensin, and calcineurin) cannot undergo peristaltic movement and develop non-obstructive hydronephrosis [20]; thus, peristaltic movement of the ureter is known to be crucial for maintaining urine flow.

Because it was challenging to reproduce smooth muscle construction and peristalsis, pulsating cardiomyocyte sheets [14] were used to provide “movement” to the Biotube. Compared to the reconstruction of the Biotube alone, the patency improved and the layered structure of the urothelium was maintained in the regenerated ureter. However, peristalsis could not be clearly observed two weeks later, and pathologically, only a few residual cardiomyocytes could be observed. This might be because, compared to the regenerated vessels [14], the area around the Biotube, especially in the center, may have become necrotic due to a lack of blood flow from the surrounding tissue.

We hypothesized that this improvement in the patency and laminar structure of the urothelium might be due to the paracrine effects of the cardiomyocyte sheets. Subsequently, experiments were conducted to investigate if the abundant paracrine effects of ASC sheets [21] can improve patency. ASC sheets are thought to be more feasible for clinical applications than cardiomyocyte sheets because of the availability of autologous cells. However, the ASC sheets did not show a supportive effect on patency or urothelial regeneration. A possible reason for this is that secreted humoral factors differ between neonatal cardiomyocytes and adult ASCs. For example, SHH, a morphogen whose downstream proteins control the layered structure of the ureter [22], is reported to be expressed in developing organs such as lungs [23]. Another possibility is that the beating of cardiomyocyte sheets in the early stages promotes urinary flow, promoting urothelial regeneration. This study shows that the presence of urinary flow may enhance urothelial intraepithelial spread.

This study has some limitations. First, although ureteral patency was achieved by adding cardiomyocyte sheets, peristalsis of the ureter could not be reproduced. Peristalsis is required when creating a substitute ureter for clinical applications. If we can facilitate the invasion of smooth muscle, stromal cells, and the nervous system into the reconstructed area and recreate peristalsis, a more physiological ureter that does not cause non-obstructive hydronephrosis could be regenerated. Second, the mechanism by which ASC sheets did not promote ureteral patency but cardiomyocyte sheets did has not yet been elucidated. The detailed underlying mechanisms require further investigation. Third, we used syngeneic rat neonates to create cardiomyocyte sheets. Unlike ASCs, autologous cells cannot be used in clinical circumstances, which is a limitation that could be overcome using human-induced pluripotent stem cells.

## 5 Conclusion

By covering autologous collagen tubes with syngeneic cardiomyocyte sheets, the patency of reconstructed ureteral lumen was improved, and the ureter was regenerated while preserving the arrangement of the urothelium. This study suggests a novel ureteral reconstruction strategy using bioengineering technologies.

## Contribution

Yamamoto, Matsui, Kinoshita, and Kobayashi wrote this manuscript.

Yamamoto, Matsui, and Saito created the hydronephrosis model.

Yamamoto, Matsui, and Sekine created the cardiomyocyte sheets.

Matsui and Sekine created the ASC sheets.

Kinoshita, Sekine, and Kobayashi performed ureteral reconstruction using a Biotube.

Matsui performed histological evaluations.

Yamamoto, Matsui, and Kinoshita performed contrast-enhanced CT evaluations.

Sasaki, Yokoo, Kimura, Kume, and Kobayashi performed supervised experiments.

All authors reviewed and approved the final manuscript.

## Funding

This study was funded by the 10.13039/501100001691Japan Society for the Promotion of Science (grant number 22H03928).

## Institutional review board statement

Animal studies were approved by the Institutional Animal Care and Use Committee and the Recombinant Gene Research Safety Committee of Jikei University School of Medicine (permit numbers: 2020-077). This study was conducted in accordance with the National Institutes of Health Guidelines for the Care and Use of Laboratory Animals.

## Data availability statement

All relevant data supporting the findings of this study are either included within the article or are available upon request from the corresponding author.

## Declaration of competing interest

The authors declare the following financial interests/personal relationships which may be considered as potential competing interests: Yasuhide Nakayama reports financial support was provided by Biotube Co Ltd. Yasuhide Nakayama reports a relationship with Biotube Co Ltd that includes: board membership and equity or stocks.
